# Supplementary material for: Identification of photoperiod-induced specific miRNAs in the adrenal glands of Sunite sheep (Ovis aries)
Source: Front Vet Sci. 2022 Jul 22;9:888207. doi: 10.3389/fvets.2022.888207 (PMC9354845; doi:10.3389/fvets.2022.888207)
Supplement: Supplementary file 1 [file Data_Sheet_1.ZIP › Supplementary file/miRDeep2 file.zip!/miRDeep2 file/Know_Structure/SPLP42A1/oar-mir-143.pdf]

miRBase precursor : oar-mir-143
Total read count : 417218
oar-miR-143 read count : 414557
remaining reads : 2661

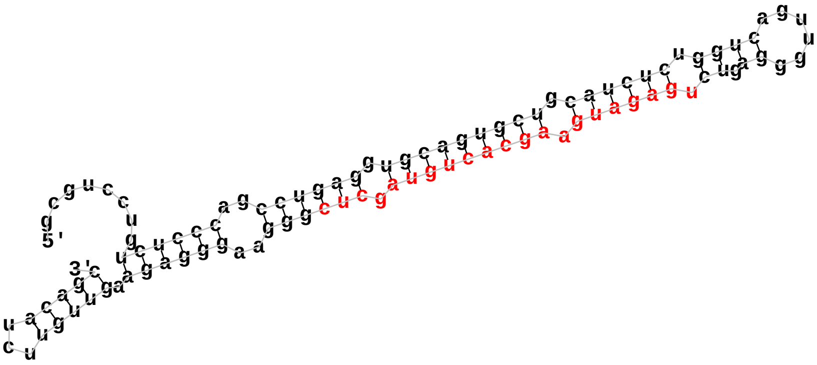

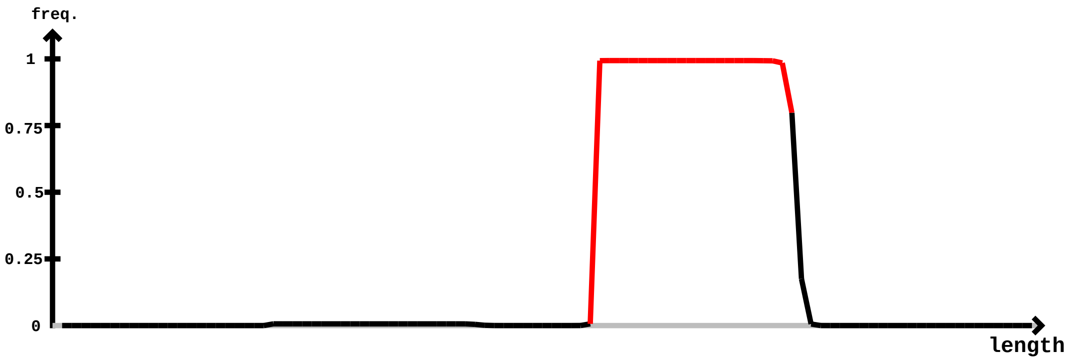


```
                                                                   oar-miR-143
5'- gcguccugucucccagccugaggugcagugcugcaucucuggucaguugggagucugagaugaagcacuguagcucgggaagggagaaguuguucuacagc  -3'    exp
    ........((((((..((((((.(((((((((.((((((.((((......)).)).)))))).))))))))).))))))..)))))).(((((...)))))    reads    mm    sample
    ....................aggugcagugcugcaucucug............................................................    2        0     seq
    ....................aggugcagugcugcaucucugg...........................................................    1        0     seq
    ....................aggugcagugcugcaucucuggu..........................................................    1        0     seq
    ....................aggugcagugcugcaucucugguc.........................................................    1        0     seq
    .....................ggugcagugcugcaucucu.............................................................    3        0     seq
    .....................ggugAagugcugcaucucu.............................................................    2        1     seq
    .....................ggugcagugcugcaucucug............................................................    8        0     seq
    .....................ggugcagugcugcaucucugg...........................................................    774      0     seq
    .....................ggugcagugcugcaucucuggu..........................................................    1401     0     seq
    .....................ggugcagugcugcaucucugguc.........................................................    424      0     seq
    .....................ggugcagugcugcaucucuggucа........................................................    38       0     seq
    .....................ggugcagugcugcaucucuggucaguu.....................................................    1        0     seq
    ......................gugcagugcugcaucucuggu..........................................................    1        0     seq
    ......................gugcagugcugcaucucugguc.........................................................    1        0     seq
    .......................................uggucaguugggagucuga...........................................    1        0     seq
    .......................................uggucaguugggagucugag..........................................    1        0     seq
    ....................................................gucugagaugaagcacuguagcu..........................    1        0     seq
    .....................................................ucugagaugaagcacuguagc...........................    4        0     seq
    .....................................................ucugagaugaagcacuguagcu..........................    8        0     seq
    .....................................................ucugagaugaagcacuguagcuc.........................    83       0     seq
    .....................................................ucugagaugaagcacuguagcucg........................    1        0     seq
    ......................................................cugagaugaagcacugu..............................    2        0     seq
    ......................................................cugagaugaagcacuguag............................    4        0     seq
    ......................................................cugagaugaagcacuguagc...........................    37       0     seq
    ......................................................cugagaugaagcacuguagcu..........................    588      0     seq
    ......................................................cugagaugaagcacuguagcuc.........................    1741     0     seq
    ......................................................cugagaugaagcacuguagcucg........................    220      0     seq
    ......................................................cugagaugaagcacuguagcucgg.......................    4        0     seq
    .......................................................ugagaugaagcacuguG.............................    13       1     seq
    .......................................................ugagaGgaagcacugua.............................    1        1     seq
    .......................................................ugagGugaagcacugua.............................    2        1     seq
    .......................................................ugagaugaagcacugua.............................    140      0     seq
    .......................................................ugagaugaagcacuUua.............................    1        1     seq
    .......................................................ugaCaugaagcacugua.............................    1        1     seq
    .......................................................ugagaugGagcacugua.............................    2        1     seq
```

oar-miR-143

gcguccugucucccagccugaggugcagugcugcaucucuggucaguugggagucugagaugaagcacuguagcucgggaagggagaaguuguucuacagc			
....ugagaugaagcGcugua.............................	1	1	seq
....ugagaugaagUacugua.............................	2	1	seq
....ugagaugaagcacuguag............................	234	0	seq
....ugagaugaagcacuguagc...........................	3266	0	seq
....ugagaugaagcacuguagcu..........................	77431	0	seq
....ugagaugaagcacuguagcuc.........................	257806	0	seq
....ugagaugaagcacuguagcucg........................	70435	0	seq
....ugagaugaagcacuguagcucgg.......................	2345	0	seq
....ugagaugaagcacuguagcucggg......................	33	0	seq
....gagaugaagcacuguagc...........................	2	0	seq
....gagaugaagcacuguagcu..........................	12	0	seq
....gagaugaagcacuguagcuc.........................	76	0	seq
....gagaugaagcacuguagcucg........................	30	0	seq
....agaugaagcacuguagcu..........................	2	0	seq
....agaugaagcacuguagcucg........................	2	0	seq
....agaugaagcacuguagcucgg.......................	12	0	seq
....agaugaagcacuguagcucggg......................	1	0	seq
....gaugaagcacuguagcuc.........................	2	0	seq
....gaugaagcacuguagcucgg.......................	6	0	seq
....gaugaagcacuguagcucggg......................	4	0	seq
....gaugaagcacuguagcucggga.....................	2	0	seq
....ugaagcacuguagcucg........................	1	0	seq
